# The Impact of the Storage Conditions and Type of Clearomizers on the Increase of Heavy Metal Levels in Electronic Cigarette Liquids Retailed in Romania

**DOI:** 10.3390/toxics10030126

**Published:** 2022-03-05

**Authors:** Alexandra Jităreanu, Irina Gabriela Cara, Alexandru Sava, Ioana Mârțu, Ioana-Cezara Caba, Luminița Agoroaei

**Affiliations:** 1 Department of Toxicology, Faculty of Pharmacy, University of Medicine and Pharmacy “Grigore T. Popa”, 700115 Iași, Romania; jitareanu.alexandra@umfiasi.ro (A.J.); ioana-cezara.caba@umfiasi.ro (I.-C.C.); luminita.agoroaei@umfiasi.ro (L.A.); 2 Research Institute for Agriculture and Environment, “Ion Ionescu de la Brad” University of Life Sciences, 700115 Iasi, Romania; 3 Department of Analytical Chemistry, Faculty of Pharmacy, University of Medicine and Pharmacy “Grigore T. Popa”, 700115 Iași, Romania; alexandru-i-sava@d.umfiasi.ro; 4 Department of Dental Technology, Faculty of Dental Medicine, University of Medicine and Pharmacy “Grigore T. Popa”, 700115 Iași, Romania; ioana.martu@umfiasi.ro

**Keywords:** e-liquids, heavy metals, storage

## Abstract

The growing popularity of electronic cigarettes has raised several public health concerns, including the risks associated with heavy metals exposure via e-liquids and vapors. The purpose of this study was to determine, using atomic absorption spectrometry, the concentrations of Pb, Ni, Zn, and Co in some commercially available e-liquid samples from Romania immediately after purchase and after storage in clearomizers. Lead and zinc were found in all investigated samples before storage. The initial concentrations of Pb ranged from 0.13 to 0.26 mg L^−1^, while Zn concentrations were between 0.04 and 0.07 mg L^−1^. Traces of nickel appeared in all investigated e-liquids before storage but in very small amounts (0.01–0.02 mg L^−1^). Co was below the detection limits. We investigated the influence of the storage period (1, 3, and 5 days), storage temperature (22 °C and 40 °C), and type of clearomizer. In most cases, the temperature rise and storage period increase were associated with higher concentrations of heavy metals. This confirms that storage conditions can affect metal transfer and suggests that the temperature of storage is another parameter that can influence this phenomenon.

## 1. Introduction

Over the past decade, electronic nicotine-delivery systems, assigned as e-cigarettes, have been viewed as a substitute with fewer health risks compared to conventional tobacco cigarettes [1,2].

The progress of these products’ technology generated a diverse range of e-cigarettes types available on the market worldwide. The generation of e-cigarettes design consists of closed- and open-system devices as described by Chen et al. [3]. Open-system devices have three fundamental items: a battery, a clearomizer, and a refillable tank where users can mix different e-liquids [3]. Typically, e-cigarettes transform a liquid solution consisting of propylene glycol, vegetable glycerol, as well as nicotine, and flavors into aerosols, which are inhaled [4,5,6,7].

The composition of e-liquids and e-cigarettes aerosol is crucial in determining potential health implications. The analysis can be challenging due to the great variety of e-liquids present on the market. Several studies identified toxicants, such as tobacco-specific nitrosamines and other nicotine decomposition products, metals, and carbonyl compounds [4,5,8].

Toxic metals, such as nickel (Ni), zinc (Zn), and lead (Pb), may be present in electronic cigarettes as well as in the aerosols formed, exposing users and those in immediate proximity (passive vaping). These metals can originate from e-liquids but mostly from the metal coils included in the clearomizer of the e-cigarette device. The Scanning Electron Microscopy Energy–Dispersive X-Ray (Sem-EDX) analysis of e-cigarette coils revealed the presence of metals, such as chromium (Cr), nickel (Ni), lead (Pb), zinc (Zn), and copper (Cu), and consequently, the transfer to the e-liquids and aerosols is possible [9,10,11,12].

Several metals, including cadmium (Cd), chromium (Cr), lead (Pb), nickel (Ni), zinc (Zn), copper (Cu), iron (Fe), and arsenic (As), have been found in e-cigarette samples and further detected in human biological samples collected from e-cigarette users. Inductively Coupled Plasma Mass Spectrometry (ICP-MS), Atomic Absorption Spectrometry (AAS), Total Reflection X-ray Fluorescence (TRXF), and Molecular Fluorescence are common techniques used to analyze heavy metals in e-cigarettes [13,14,15]. Therefore, with the exception of Cd, similar metals’ concentrations were found in the biological samples collected from e-cigarette users compared with conventional tobacco cigarette smokers [16]. Although cobalt (Co) is not a common element found in the environment or in the composition of alloys used in the construction of e-cigarettes or other ENDS (electronic nicotine delivery systems), small amounts of this metal were identified in the components of clearomizers from all generations [17].

The longer-term effects of e-cigarettes exposure are still inconclusive, but the existing literature reports revealed their inflammatory, irritant, and cytotoxic potential [18]. The major route of metal exposure is through direct or secondary inhalation of e-liquids, which is associated with serious health threats, such as carcinogenic and neurotoxic risks [19]. The risks are augmented by the size of the particles. E-cigarette aerosols contain nanoparticles (11–25 nm median diameter) and submicron particles (96–175 nm median diameter) [20]. The size of the inhaled particles is important for the depth of airway penetration, and the toxic potential can be enhanced by the high penetration of small-sized particles in tissues and organs [21,22]. Re et al. found a connection between chronic e-cigarette aerosol exposure and endogen metal dyshomeostasis, which has been linked to the onset of neurodegenerative diseases, such as Alzheimer’s and Parkinson’s [19]. The risk of neurotoxicity is significantly higher for young people’s developing brains. They proved that neurotoxic levels of metals accumulated in the striatum, the frontal cortex, and the ventral midbrain of rodents after exposure to e-cigarette aerosols, increasing the risks of developing neurological disorders and neurodegenerative diseases [19]. Metal accumulation in the nervous system in the case of e-cigarette use is enhanced by the alteration of the blood–brain barrier integrity [23].

Long-term Pb exposure could be related to a variety of neurological and peripheral structure illnesses, cardiovascular issues, and muscle system abnormalities in humans [24]. Chronic inhalation of lead nanoparticles is associated with cardiovascular, respiratory, and central nervous system alterations. The results of the studies concerning lead exposure for e-cigarette users are still contradictory. Wiener and Bhandari found similar blood lead levels in subjects who used or did not use e-cigarettes, while Goniewicz et al. showed that the urinary level of lead was lower in never-users than in e-cigarette smokers [25,26]. In a study performed on 100 participants, Olmedo et al. evaluated exposure to metals through e-cigarettes by assessing the metal levels in non-invasive biological samples (urine, hair, and exhaled breath condensate) [27]. Metals such as Cr, Cu, Pb, and Sn were found in higher quantities in the urine of e-cigarette users, but the study could not correlate the metal levels in the biological samples with the concentrations determined in e-vapors. It could not confirm that vaping was the main source of metal exposure [27].

Ni is a toxic metal, and its adverse health effects are linked to changes in heart rate, oxidative stress, and the consequent lung, nasal, and paranasal cancers [9,28,29]. The possible toxic effects of e-cigarette are also related to respiratory system damage. Ni is classified as inhalation carcinogens, and the lung represents the most sensitive target of Ni toxicity [16,30]. The results of Fowles et al. estimated the toxicity of heavy metals (especially chromium and nickel) in e-liquids and aerosols and related to major health issues, such as cancer [24]. The prolonged exposure of Ni in the human body can significantly increase the risk of cancer [24].

Another metal of concern is Co. Cobalt exposure can cause hematopoietic effects, cardiomyopathy, hypothyroidism, and thyroid hyperplasia, and it also has irritant effects on the respiratory tract [31]. A recent study investigated the association between cobalt exposure (cobalt lung) and e-cigarette users who developed giant cell interstitial pneumonia and hard metal pneumoconiosis [32], but several inconsistencies were identified in this report (cobalt was not determined in the original method cited by the authors, and Co was not found in the lung samples collected from the patient) [33].

In accordance with its function to human growth and development, Zn is one of the more fundamental elements and a cofactor for the activity of many enzymes, but inhaling large amounts of Zn and Zn-derivative nanoparticles can cause airway inflammation [16,34]. Increased Zn concentrations have been associated with copper deficiencies in the liver and heart along with metalloenzymes function interference and iron storage, resulting in anemia [35]

Several parameters were investigated to see their influence on metal concentrations in both e-liquids and aerosols. Zhao et al. determined the concentrations in e-cigarette aerosols produced in open- and closed-systems devices and concluded that the device type influenced metal release to aerosols; aerosols generated in open-system devices presented higher concentrations of metals [1]. Furthermore, metal concentrations increased with power setting, and a higher voltage is associated with an increased coil temperature and a higher probability of degradation and metal emissions. Differences in coil composition can also affect metal levels in aerosols [1].

In some cases, the e-liquids can remain in clearomizers for several days, stored at different environmental temperatures, and it is important to identify the factors that influence metal emissions of the components of the clearomizers. Na et al. investigated the metal release phenomenon during storage and use [11]. They concluded that metal transfer is influenced by the duration of storage in the e-cigarette device and that the concentrations of heavy metals found in e-liquids were significantly higher after e-cigarette use [11].

Starting from these findings, the present study aimed to determine the concentration of some important heavy metals (Pb, Ni, Zn, and Co) in some e-liquids found on the Romanian market. Samples from five (5) different e-cigarette brands were obtained from national retail markets. The heavy metal content after purchasing (from e-liquid bottles) and storage period (1, 3, and 5 days) at different temperatures (22 °C and 40 °C) were analyzed, and their concentrations were linked to World Health Organization (WHO) and Food and Agriculture Organization (FAO) recommended limits.

## 2. Materials and Methods

### 2.1. Sample Preparation

The types of electronic cigarette samples (ECS) were purchased from the national market outlets (from VapePoint and Etigareta shops, Iasi, Romania). A total of five commercially available e-liquid samples of various nicotine concentrations and different flavoring agents were selected for this study (Table 1). The samples were selected randomly, but the variable nicotine concentration and the different flavor and propylene glycol:vegetable glycerin ratio were taken into consideration for the selection. The packaging of the liquids consisted of 10 mL plastic dropper bottles (the dropper lids were also made from plastic). The samples coded from A to E were kept at room temperature (22 °C) until analysis. The basic composition description of the EC liquids (according to the manufacturer) consists predominantly of propylene glycol (PG) and vegetable glycerin (VG).

**Figure 1:**
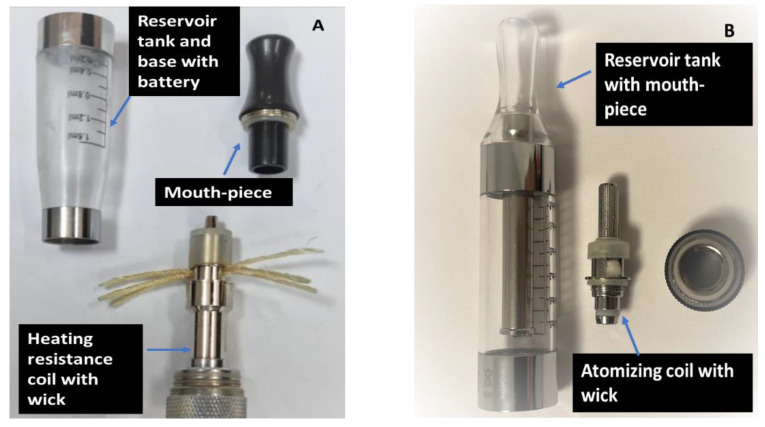
Anatomy of the clearomizers used in the experiment ((**A**) clearomizer 1; (**B**) clearomizer 2).

For each sample, their heavy metal content was analyzed under three variables/conditions:

- I: The initial phase: the EC liquids were directly taken from EC liquid bottles as purchased from retail;
- II: EC liquid analyzed for storage period and clearomizer effect: the samples were stored for 1, 3, and 5 days in 2 different types of EC clearomizers purchased from specialized shops (VapePoint and Etigareta shops, Iasi, Romania). The clearomizers were selected based on their popularity. According to the employees from the vape shops, at the time of the purchase, these models were requested most frequently by the customers. Both clearomizers were “tank-style” electronic cigarettes and belonged to the second generation of electronic cigarettes [36,37]; clearomizer 1 was a CE4 type, while clearomizer 2 was a T3S type. The clearomizers (Figure 1) presented different tank capacities (1.6 mL and, respectively, 3.0 mL) and were made from dark plastic material (clearomizer 1) and clear, resistant plastic (clearomizer 2). Inside the tank, an atomizing unit with metallic coil and wick material were visible. The prices for the two clearomizers were also different (rating as ‘’low’’—clearomizer 1 and ‘’high’’—clearomizer 2);
- III: EC liquid analyzed for storage period and temperature effect: EC liquids were stored in the two clearomizers mentioned above at two different temperatures: 22 °C (room temperature) and 40 °C. In this step, the samples were maintained in room with controlled temperature (22 °C), in the absence of direct sunlight, and in a programmable furnace (Model Nobertherm, Germany) at 40 °C for 1, 3, and 5 days in order to investigate the concentration of heavy metals that can be released through their storage under improper/inadequate conditions. For each clearomizer and both the variables (storage period and temperature), three replicates of each sample were performed. The clearomizers were filled and sealed with the e-liquid, from which an aliquot of 1 mL was separated and analyzed.

The storage period and temperature were chosen in order to mimic real-life scenarios. Electronic cigarette users do not keep e-liquids inside the clearomizers for more than a few days before using them, and that is why we chose a five-day limit for the storage period. The room temperature is usually around 22 °C, but it can reach 40 °C during very hot summer days; we have chosen these two temperature values to evaluate the storage temperature’s influence on metal transfer.

For heavy metal analysis, 1 mL of each e liquid sample was performed by diluting with 10 mL of 5% HNO_3_ solution. This mixture was sonicated for 30 min (Elma S180, Elmasonic sonicator), and then, the solution were analyzed by AAS [11]. A blank e-liquid sample was prepared by mixing PG and VG at the same ratio (1:1, *w/w*) and analyzed according to real sample method.

### 2.2. Reagents and Standards

All reagents and chemicals used in this study were of analytical grade. Nitric acid (HNO_3_ Suprapur 65%, Merck, Darmstadt, Germany) and mono-element containing stock standard solutions of Ni, Pb, Zn, and Co (1000 mg L^−1^, Merck, Darmstadt, Germany) were used to obtain the standard solution for the calibration curve.

Calibration standards were prepared by diluting the primary standard with 5% HNO_3_ at five different concentration levels (0.05, 0.1, 0.2, 0.25, and 0.5 mg L^−1^). All dilutions were performed using high-purity deionized water obtained from a Milli-Q water purification system (Millipore, Bedford, MA, USA).

The samples were prepared in 25 mL glass flasks (class A), which were previously immersed in 1% HNO_3_ warm aqueous solution for at least 6 h and then rinsed with ultrapure water.

### 2.3. Instrument

An atomic absorption spectrometer-AAS (ContrAA 700, Analytikjena, Jena, Germany) was conducted to assess the metals concentrations. The parameters that were used to determine the concentration of heavy metal by AAS were a high-resolution continuum source, equipped with a xenon short lamp with UV arc in hot spot mode and a high-resolution echelle grating monochromator. The flame was generated using an air-acetylene mixture with 99.95% purity.

Accuracy, linearity, precision, limit of detection (LOD), and limit of quantification (LOQ) are some of the analytical criteria used to validate the optimized method.

The correlation coefficient (R^2^) of the calibration curves was used to calculate the linearity. As part of the instrument’s performance and method accuracy, the recovery of standard spiked samples was assessed using 5% HNO_3_ method [11]. It was performed at each stage by spiking the e-liquid samples with two different concentrations (1.0 and 5.0 mg L^−1^) of a mono-element standard. A blank sequence and spiked blanks were performed at each stage to ensure the results and cancel the matrix interferences.

The relative standard deviation (RSD) of the triplicate measurements of each e-liquid sample was used to compute the precision value. As a result, the values of this procedure are reported as an average RSD of triplicate measurements.

The limit of detection (LOD) was the lowest amount of metal that can be detected and was estimated by dividing the SD of three measurements of the PG/VG mixture with the slope of the calibration curve. The limit of quantification (LOQ) was defined as the smallest amount that can be quantitatively identified at a specified precision and accuracy.

### 2.4. Data Analysis

Three replicates were taken for each sample, and the average value was calculated. The mean values were statistically analyzed using the *t*-test with a 95% confidence level.

## 3. Results

### 3.1. Calibration and Detection Limit

Table 2 presents the calibration results for the determination of heavy metals Pb, Zn, Ni, and Co by AAS technique. The correlation coefficient was used to confirm the linearity of each trace element (R^2^). The concentration ranged between 0.05–0.50 mg L^−1^ was established among absorbance and metal concentration; all calibration curves showed good linearity (R^2^ > 0.997). The obtained LOD values ranging between 0.001–0.04 mg L^−1^ highlights the sensitivity of the method, as the analytical parameters are low compared with other analytical techniques [38].

PG/VG mixture and e-liquids’ samples spiked with concentration of 1.0 and 5.0 mg L^−1^ using mono-element standard registered 94.8 to 101% and 94.1 to 107.3% of the recoveries, with RSD less than 20% at all spiked quantities (Table 3). The method’s accuracy was found to be appropriate and was confirmed for each heavy metal through real and spiked values measured in comparison.

The recoveries for the reliability assessment of our experimental method based on spiked samples ranged between 94–107% with relative standard deviation ranged between 1.7–7.8%. According to these findings, the method presents good performance characteristics.

### 3.2. Heavy Metals Concentration in E-Cigarettes

The results of the heavy metal analysis using AAS for e-cigarette items being sold in Romania markets are presented. Consequently, the five e-cigarette brands were discovered to contain quantifiable levels of heavy metals (Table 4).

#### 3.2.1. Lead Concentration

In the present study, lead was found in all investigated samples before storage. The initial mean values of this metal ranged from 0.13 to 0.26 mg L^−^^1^. The highest concentration of Pb was exhibited by sample C.

**Figure 2:**
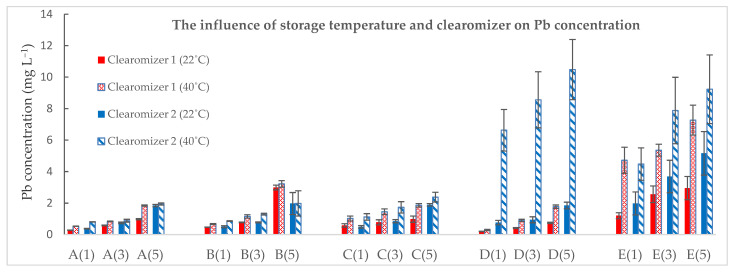
The influence of storage temperature and of the clearomizer on Pb concentration. Sample code: Sample number (number of storage days in the clearomizer).

In Figure 2 are shown the Pb concentrations obtained for the five samples and their variation under different experimental conditions.

As the storage period increased (from 1 to 5 days), the reported values in the five E-liquid samples for Pb also tended to increase. This pattern of Pb concentration was found in both types of clearomizers after storage, which showed that there are significantly higher differences after storage than the initial ones at the 0.05 level (*p*-value < 0.05) (Table 5).

In addition, the current study investigated the influence of temperature on Pb transfer after storage in the two types of clearomizers (Figure 1). After increasing the storage temperature from 22 °C to 40 °C, higher Pb concentrations in the e-cigarette samples were obtained. The Pb content found in e-liquids sample E (after storage in both clearomizers) and sample D (after storage in clearomizer 2) showed the greatest increase, which suggests the release and transfer of heavy elements from the metal substrates of different components of clearomizers. The statistical analysis regarding the influence of temperature on Pb transfer was performed using the *t*-test. For clearomizer 1, the results of the analysis showed a significant difference at the 0.05 level only for samples B, C, and E, while for clearomizer 2, the results were significantly different at the 0.05 level for samples C, D, and E.

The research also included a comparison of the clearomizer type on Pb transfer when stored at temperatures of 22 °C and 40 °C. The results obtained were heterogeneous; for e-liquid samples A, C, D, and E, the average transfer of Pb was higher after storage in clearomizer 2, while for sample B, the transfer was higher for clearomizer 1.

The statistical analysis regarding the influence of the clearomizer on Pb transfer was performed using the *t*-test. At 22 °C, the results showed a significant difference at the 0.05 level only for samples D and E, while after storage at 40 °C, there was a significant difference only for sample D.

#### 3.2.2. Nickel Concentration

The present study recorded small concentrations of Ni (0.01–0.02 mg L^−1^) in each investigated EC liquid sample before storage. The related values for Ni content in the EC liquids are presented in Table 4.

**Figure 3:**
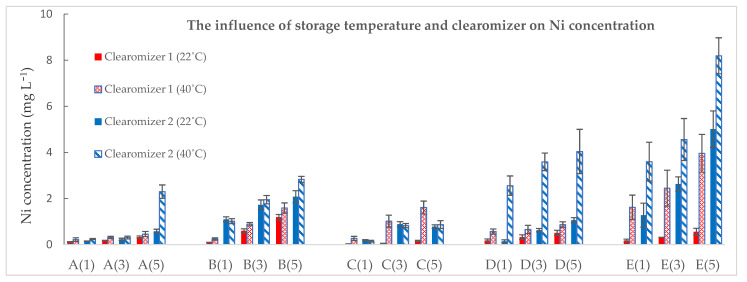
The influence of storage temperature and of the clearomizer on Ni concentration. Sample code: Sample number (number of storage days in the clearomizer).

As a general trend, storage at a higher temperature (40 °C in comparison to 22 °C) increased Ni transfer. The ascending trend of Ni levels in e-liquids in relation to the storage period and temperature is visible in Figure 3.

According to our results, the concentrations of Ni were higher after the storage period, sustaining the possible metal transfer from the metallic parts of the clearomizer to the solutions. It is likely that Ni concentrations increased after storage in both clearomizers, with values significantly greater than the initial ones at the 0.05 level (Table 6).

The statistical analysis regarding the influence of temperature on the metal transfer was performed using the *t*-test. For clearomizer 1, the results of the analysis showed a significant difference at the 0.05 level for all samples, while for clearomizer 2, the results were significantly different at the 0.05 level only for samples D and E.

When analyzing the influence of the clearomizer (Figure 2), the data suggested that Ni transfer was more pronounced in the case of the EC liquids stored in clearomizer 2. The statistical analysis was performed using the *t*-test. At 22 °C, the results of the analysis showed a significant difference at the 0.05 level for samples B, C, and E, while at 40 °C, the results were significantly different at the 0.05 level for samples B, D, and E (*p* < 0.05).

#### 3.2.3. Zinc Concentration

The concentrations of Zn in EC-liquid samples established during the present study are indicated in Table 4.

Concentrations of Zn were identified in EC liquid samples before storage. Zn initial concentrations were lower than the determined concentration of lead and ranged between 0.04–0.07 mg L^−1^.

**Figure 4:**
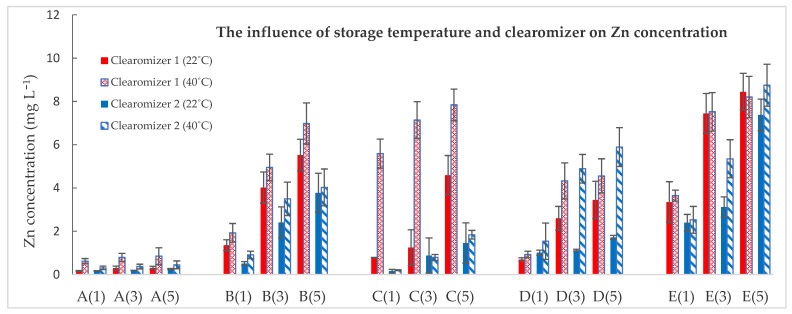
The influence of storage temperature and of the clearomizer on Zn concentration. Sample code: Sample number (number of storage days in the clearomizer).

The concentrations of Zn increased significantly according to the storage period (from 1 to 5 days), and also, high levels of Zn were associated with storage at 40 °C temperature (Figure 4).

In several samples, the amount of Zn as a result of the storage period in both types of clearomizers was more than 100 times higher, with statistically significant differences at the 0.05 level (Table 7). Moreover, our findings were very comparable to those of other studies all supporting the claim that heavy metals can be transported to the liquids via EC devices [11,39].

The *t*-test analysis was performed for the influence of temperature on zinc transfer. For clearomizer 1, the results of the analysis showed a significant difference at the 0.05 level for samples A, B, and C, while for clearomizer 2, the results were significantly different at the 0.05 level only for sample A. Moreover, we found that the two types of clearomizers released different amounts of metals when the same temperature (40 °C) was used; while the concentrations of Ni released was more powerful after storage in clearomizer 2, Zn concentration tended to be higher after storage in clearomizer 1 (Figure 3). In addition, the *t*-test analysis showed at 22 °C no significant difference, while at 40 °C, a significant difference at the 0.05 level was found for samples A, B, C, and D.

#### 3.2.4. Cobalt Concentrations

Cobalt levels were in most cases below the LOD even as the period of storage and temperature increased. More data are needed to evaluate the significance of e-liquids as exposure sources for Co.

## 4. Discussion

The systemic toxicity of heavy metals has become a subject of great interest in the last few years. Several research groups investigated this phenomenon and the factors that influence the levels of heavy metals in e-liquids and aerosols produced during vaping. The e-liquids are able to heighten the heavy metal content depending on the manufacturing material and design of the used devices but also in relation to their composition (the ratio of propylene glycol to glycerol, nicotine, pH modifiers, and different flavors) [10,40]. The quality of the constituents (propylene glycol, glycerol, nicotine, and flavors) can be important because they can be a source of heavy metals. A study conducted by Kamilari et al. found high levels of Cd and Ni in the nicotine and two flavoring agents used for the production of e-liquids [15].

Another key element is the electronic device itself. Palazzolo et al. also found higher concentrations of metals (e.g., Ni and Zn) in the aerosols produced during vaping than in the e-liquids, pointing to the electronic cigarette device as the source of the metals [14]. They indicated the source for Ni to be, most likely, the core tip, the resistance coil, and the wiring and welding within the core assembly [14]. The analysis performed on the elemental composition of clearomizers revealed that the materials used included metals, such as chromium, nickel, tin, zinc, and copper, and that the components in these devices were very similar regardless of the brand and generation [41]. As Olmedo et al. pointed out, the spike of metal levels (like nickel) in e-liquid samples after they were exposed to the heating element suggests that heating coils are a potential source of the metals [39,42]. When electrical power is applied, the heating coils can produce metallic nanoparticles, which can condense and coagulate into nanoparticle clusters. Wilson et al. analyzed the characteristics of metallic nanoparticles generated by the heating of an electronic cigarette coil in the absence of a nicotine solution [43]. According to their results, using a low-resistance coil can reduce metal exposure [43]. Modifying the electronic devices’ designs and using materials of suitable quality are ways for lowering the concentration of potentially dangerous metals in e-liquids and e-vapors [42,44].

A research subject imminently related to the assessment of the levels of heavy metals in e-liquids (and the understanding of the various factors that can influence these concentrations) is to evaluate to what extent heavy metals are transferred from e-liquids to aerosols. Previous studies revealed that the transfer of heavy metals to aerosols is not uniform; it depends on the different topographies for aerosol production (puffing protocol), but the results obtained are especially sensitive to the efficiencies of the methods of aerosol collection [36]. Thus far, several methods were reported for e-cigarette aerosol collection in the literature [36,45,46], but the absence of a standardized procedure makes it difficult to evaluate the real quantity of metals delivered through e-cigarette aerosols and to estimate potential health effects.

Our study investigated the concentrations of some heavy metals (Pb, Ni, Zn, and Co) in e-liquid samples, but we focused more on evaluating the impact of the storage conditions and type of clearomizer on the increase of heavy metals content in e-liquids.

In the case of lead, the initial concentrations (determined in e-liquids prior to the contact with the clearomizers) varied between 0.13 and 0.26 mg L^−1^. In their review, Zhao et al. pointed out that different studies reported metal levels in different ways, and for easy comparison, they recommended the conversion to the weight/weight basis using a value of 1:16 g/mL for the density of e-liquids [16]. If this algorithm of conversion would be used for our results, the values obtained would be in the range 0.11 to 0.22 mg kg^−1^. No regulations regarding heavy metals content were established until now, but JECFA (Joint FAO/WHO Expert Committee on Food Additives) adopted a general limit of 2 mg kg^−1^ for lead and a limit of 1 mg kg^−1^ or lower in case of high consumption [47]. Similar studies about the transfer characteristics of heavy metals in EC liquids reported average values of Pb between 0.12–0.25 mg kg^−1^ for the e-liquid samples analyzed before placement in the electronic cigarette device [11], while a study from Canada and the United States conducted by Dunbar et al. evaluated the heavy metals levels in e-liquids samples bottled in individual containers and in e-liquids that were extracted from inside disposable electronic devices [48]. They reported that the Pb concentrations in bottled e-liquids were not detectable above the limit of quantitation of 0.0091 mg L^−1^ (9.1 ppb) [48]. Furthermore, Olmedo et al. determined the concentrations of metals from both the e-liquids directly from the refilling dispenser (without contact with the coil) and from the tanks after the device was used [39]. They determined a value of 0.476 μg kg^−1^ (0.000476 mg kg^−1^) for the median concentration of Pb in 56 e-liquid samples in the absence of the previous contact with the electronic device [39].

Our results showed a direct link between the storage period in clearomizers and the Pb levels. These results are in agreement with the reported results of a study conducted by Na et al. [11]. They also found that the average concentration of Pb significantly increases after storage in the clearomizer [11].

In the case of nickel, the initial concentrations found in e-liquids were very low. At present, there are no maximum contaminant levels for heavy metals in e-liquids, but the European Food Safety Authority (EFSA) analyzed the risks to public health related to the presence of nickel in food and drinking water and reported the limit values established for Ni by different international organisms [49]. A value of 20 µg/L (0.02 mg L^−1^) for nickel was set in Council Directive 98/83/EC, while the WHO established a limit value 70 µg nickel/L and a tolerable daily intake (TDI) of 11 µg nickel/kg b.w. [49,50,51]. All our results did not exceed those limits.

Some of the previous studies have also reported traces of concentrations of Ni in EC liquids. Na et al. reported values of Ni in the investigated samples (represented by EC liquid bottles directly analyzed as purchased from retail) below the detection limit, 0.04 mg kg^−1^ [11]. Kamilari et al. used Total Reflection X-ray Fluorescence Spectrometry for the quantification, and the Ni concentrations found in the 22 analyzed samples varied between 0.002 and 0.017 μg g^−1^ (mg kg^−1^) [15]. For easy comparison between our results and the finding from other studies, a conversion to mg kg^−1^ could be performed using the formula of the density and considering the value of 1.16 g/mL for the density of e-liquids as recommended by Zhao et al. [16].

E-cigarette devices have a metallic coil, which heats the e-liquid generating the aerosol; these metallic coils are manufactured with nickel (Ni) and chromium (Cr) alloys, which can be released to the e-liquids during the storage and heating process [9,29]. Other data support that the nichrome from the heating elements is resistant to oxidation (even at high temperatures) and suggest other alloys as possible sources of chromium, iron, and nickel oxide. However, the authors cannot exclude a limited degree of degradation of nichrome heating elements caused by extensive use periods [21].

The Ni levels increased after storage in the clearomizers. The results of the current study are in accordance with the findings of Na et al. [11]. This means that the clearomizer composition and conditions influenced the concentration of heavy metals. Thus, even without the heating procedure, the majority of the metallic components in the liquids are enhanced just by being stored in the clearomizer. Olmedo et al. determined a value of 2.03 μg/kg (0.0023 mg kg^−1^) for the median concentration of Ni in e-liquids sampled directly from the refilling dispenser (without contact with the coil), while in the liquids after puffing the e-cigarettes, the Ni median concentration was 100 times higher [39].

In the case of zinc, the Joint FAO/WHO Expert Committee on Food Additives established a provisional maximum tolerable daily intake (PMTDI) of 1.0 mg/kg of body weight [52]. The Zn levels determined in the e-liquids after purchase ranged from 0.04–0.07 mg L^−1^. If we applied the conversion algorithm mentioned by Zhao et al., our results would range between 0.03–0.06 mg kg^−1^. We can state that the results of the current research are in accordance with previously published results of Na et al., which determined the heavy metals concentrations in e-liquids directly taken from bottles as purchased from retail [11]. They reported Zn concentrations between 0.05–0.63 mg kg^−1^ [11]. In the samples they analyzed, Olmedo et al. obtained a median concentration of 13.1 μg/kg (0.0131 mg kg^−1^) without previous contact with the device [39]. Another study conducted by Gray et al. reported a connection between the higher Zn concentration in samples from devices with brass electrical connectors [10].

In the case of cobalt, a limit value of 0.1 mg kg^−1^ (per day) was identified to be the reliable dose of the substance for non-toxic level (NOAEL), while for chronic exposure via inhalation, a MRL of 1 × 10^−4^ mg m^−3^ was set by ATSDR (The Agency for Toxic Substances and Disease Registry) [53,54]. In our samples, the cobalt levels were below the detection limit (0.005 mg L^−1^).

In our study, sample 5 had the highest concentration of nicotine (18 mg/mL) and a ratio of propylene glycol:glycerol of 50:50, and it presented the highest values for Pb, Ni, and Zn after storage in both clearomizers. A study conducted by Zervas et al. concluded that the transfer of Fe, Ni, Cu, Zn, and Pb increased with nicotine concentration and that glycerol also facilitated metal transfer in comparison to propylene glycol [55]. However, in our case, the investigation of more samples is needed to fully support this statement.

Looking at the behavior of the five e-liquids in the two clearomizers, we observed that lead and nickel transfer were more pronounced after storage in clearomizer 2, while zinc levels increased more after the storage of the e-liquids in clearomizer 1. The most likely explanation is the differences in the design and the materials used for the electronic devices, but we can only support this statement using the results of other research [29,36,44].

This study is the first to determine the concentrations of heavy metals in e-liquids marketed in Romania, but its major limitation is the low number of samples analyzed. From these preliminary results, there is no indication of low-quality counterfeit products sold in Romania, but more extensive research is needed in the future to conclusively evaluate the safety of these products for Romanian consumers. The findings in the present paper also emphasized and confirmed that the levels of heavy metals are greatly influenced by simply storing the e-liquids clearomizers and that storage conditions can also influence this transfer process. The storage temperature (investigated for the first time, to our knowledge) is another parameter that can influence metal transfer from the components of the clearomizer to the e-liquid inside.

## 5. Conclusions

The concentrations of four heavy metals (Pb, Ni, Zn, and Co) with potentially serious implications for human health were estimated in five e-liquid samples purchased from the Romanian market. The initial concentrations were reduced in the analyzed e-liquids and increased after their storage in clearomizers at different temperatures for various periods. Co was found to be non-detectable in all the stages of the study. These findings support and consolidate the idea that heavy metals are transferred to e-liquids through the direct contact between the e-liquids and the metallic components of the devices. Heavy metals transfer depends on the characteristics of the electronic device and the composition of the e-liquids (as revealed by previous studies) but also on the storage conditions. Longer periods of storage inside the clearomizer were associated with higher levels of metals in e-liquids. Besides the period of storage, we also pointed out that storage temperature (22 °C vs. 40 °C) can also affect metal transfer from the parts of the clearomizers. This is an important aspect that needs to be taken into consideration by the manufacturers and regulatory agencies, which can introduce new recommendations that could reduce metal release during storage. Furthermore, an interesting subject for future research would be the investigation of the combined influence of certain chemical composition parameters of e-liquids and different storage conditions.

## Figures and Tables

**Table 1:** Basic composition of EC liquids selected in this study.

Sample	Nicotine (mg/mL)	PG:VG Ratio (*w/w*)	Flavor
A	0	50:50	Dark tobacco
B	6	70:30	Cherry
C	12	50:50	Apple
D	18	70:30	Tobacco
E	18	50:50	Cuban cigar

Data presented were available on the labels of the products.

**Table 2:** Calibration results for the determination of heavy metals in e-cigarettes.

Metal	Wavelength (nm)	Linear Range (mg L^−1^)	Detection Limit (mg L^−1^)	Correlation Coefficient (R^2^)	RSD (%)
Pb	217.00	0.1–0.5	0.04	0.998	1.9
Zn	213.85	0.05–0.5	0.001	0.999	2.1
Ni	232.00	0.05–0.5	0.01	0.999	1.7
Co	240.72	0.05–0.5	0.005	0.997	3.4

The measurements were done in triplicate; RSD, Relative Standard Deviation of the triplicate measurements.

**Table 3:** The average recovery (%) and RSD (%) of spiked samples.

Metal	E-Liquid Sample				PG/VG Mixture			
	1.0 mg L^−1^		5.0 mg L^−1^		1.0 mg L^−1^		5.0 mg L^−1^	
	Recovery (%)	RSD (%)	Recovery (%)	RSD (%)	Recovery (%)	RSD (%)	Recovery (%)	RSD (%)
Pb	94.1	2.5	96.2	1.9	95.7	7.8	95.9	4.1
Zn	98.3	4.5	107.3	2.1	94.8	4.6	95.5	6.9
Ni	95.4	1.7	95.7	2.3	98.1	3.9	101.3	4.8
Co	96.4	5.5	104.2	3.4	95.7	4.2	98.1	2.4

RSD, Relative Standard Deviation of the triplicate measurements; PG, propylene glycol; V, vegetable glycerin.

**Table 4:** Heavy metals (Pb, Ni, Zn) concentrations in EC liquids under different conditions.

Sample	Initial Conc. (mg L^−1^)	Clearomizer 1		Clearomizer 2	
		22 °C (mg L^−1^)	40 °C (mg L^−1^)	22 °C (mg L^−1^)	40 °C (mg L^−1^)
Pb					
A(1)	0.17 ± 0.02	0.28 ± 0.01	0.53 ± 0.01	0.37 ± 0.02	0.81 ± 0.01
A(3)		0.58 ± 0.03	0.84 ± 0.02	0.74 ± 0.05	0.88 ± 0.09
A(5)		0.98 ± 0.04	1.84 ± 0.04	1.84 ± 0.07	1.94 ± 0.07
B(1)	0.15 ± 0.03	0.47 ± 0.01	0.67 ± 0.03	0.50 ± 0.06	0.86 ± 0.02
B(3)		0.78 ± 0.02	1.15 ± 0.11	0.78 ± 0.03	1.30 ± 0.06
B(5)		2.99 ± 0.15	3.22 ± 0.21	1.97 ± 0.7	1.98 ± 0.8
C(1)	0.26 ± 0.06	0.58 ± 0.11	1.02 ± 0.16	0.49 ± 0.08	1.12 ± 0.21
C(3)		0.78 ± 0.16	1.45 ± 0.18	0.85 ± 0.10	1.73 ± 0.36
C(5)		0.98 ± 0.20	1.86 ± 0.11	1.89 ± 0.08	2.38 ± 0.31
D(1)	0.13 ± 0.01	0.20 ± 0.01	0.29 ± 0.04	0.76 ± 0.14	6.63 ± 1.32
D(3)		0.42 ± 0.03	0.90 ± 0.07	0.94 ± 0.19	8.56 ± 1.78
D(5)		0.74 ± 0.04	1.77 ± 0.11	1.85 ± 0.21	10.48 ± 1.91
E(1)	0.19 ± 0.01	1.20 ± 0.19	4.72 ± 0.83	1.98 ± 0.73	4.48 ± 1.03
E(3)		2.56 ± 0.53	5.36 ± 0.38	3.69 ± 1.03	7.88 ± 2.11
E(5)		2.95 ± 0.74	7.27 ± 0.95	5.16 ± 1.38	9.23 ± 2.18
Ni					
A(1)	0.01 ± 0.01	0.11 ± 0.02	0.22 ± 0.08	0.15 ± 0.01	0.25 ± 0.02
A(3)		0.18 ± 0.01	0.31 ± 0.05	0.23 ± 0.05	0.31 ± 0.06
A(5)		0.33 ± 0.05	0.46 ± 0.11	0.58 ± 0.09	2.30 ± 0.29
B(1)	0.02 ± 0.01	0.09 ± 0.01	0.25 ± 0.05	1.10 ± 0.11	1.02 ± 0.11
B(3)		0.60 ± 0.09	0.89 ± 0.07	1.73 ± 0.21	1.95 ± 0.18
B(5)		1.19 ± 0.12	1.59 ± 0.22	2.08 ± 0.26	2.83 ± 0.13
C(1)	0.02 ± 0.01	0.03 ± 0.01	0.27 ± 0.09	0.19 ± 0.03	0.15 ± 0.04
C(3)		0.05 ± 0.01	1.02 ± 0.26	0.89 ± 0.11	0.83 ± 0.09
C(5)		0.16 ± 0.03	1.61 ± 0.28	0.78 ± 0.08	0.87 ± 0.17
D(1)	0.01 ± 0.01	0.18 ± 0.07	0.57 ± 0.11	0.15 ± 0.06	2.56 ± 0.42
D(3)		0.33 ± 0.1	0.66 ± 0.18	0.63 ± 0.07	3.59 ± 0.38
D(5)		0.51 ± 0.11	0.87 ± 0.12	1.06 ± 0.11	4.04 ± 0.96
E(1)	0.02 ± 0.01	0.18 ± 0.06	1.62 ± 0.53	1.28 ± 0.52	3.60 ± 0.84
E(3)		0.32 ± 0.09	2.45 ± 0.78	2.63 ± 0.31	4.56 ± 0.91
E(5)		0.56 ± 0.15	3.96 ± 0.82	5.01 ± 0.79	8.19 ± 0.78
Zn					
A(1)	0.04 ± 0.01	0.17 ± 0.02	0.62 ± 0.12	0.17 ± 0.01	0.31 ± 0.08
A(3)		0.30 ± 0.09	0.79 ± 0.19	0.19 ± 0.01	0.37 ± 0.11
A(5)		0.31 ± 0.07	0.85 ± 0.39	0.25 ± 0.03	0.45 ± 0.18
B(1)	0.07 ± 0.01	1.36 ± 0.25	1.93 ± 0.43	0.91 ± 0.09	0.51 ± 0.17
B(3)		4.02 ± 0.72	4.95 ± 0.61	3.50 ± 0.72	2.41 ± 0.77
B(5)		5.52 ± 0.73	6.98 ± 0.95	4.03 ± 0.90	3.78 ± 0.85
C(1)	0.05 ± 0.01	0.78 ± 0.01	5.59 ± 0.67	0.16 ± 0.08	0.20 ± 0.02
C(3)		1.25 ± 0.82	7.14 ± 0.85	0.88 ± 0.81	0.79 ± 0.14
C(5)		4.59 ± 0.91	7.84 ± 0.73	1.46 ± 0.93	1.84 ± 0.20
D(1)	0.07 ± 0.01	0.69 ± 0.09	0.93 ± 0.15	1.01 ± 0.12	1.54 ± 0.84
D(3)		2.60 ± 0.55	4.33 ± 0.83	1.12 ± 0.05	4.89 ± 0.66
D(5)		3.45 ± 0.86	4.56 ± 0.79	1.72 ± 0.09	5.89 ± 0.90
E(1)	0.06 ± 0.01	3.35 ± 0.94	3.65 ± 0.25	2.40 ± 0.38	2.53 ± 0.62
E(3)		7.45 ± 0.92	7.52 ± 0.89	3.12 ± 0.47	5.35 ± 0.88
E(5)		8.45 ± 0.85	8.20 ± 0.96	7.38 ± 0.73	8.75 ± 0.97

Data are presented as mean ± SD (standard deviation). Sample code: Sample number (number of storage days in the clearomizer).

**Table 5:** The influence of the duration of storage on Pb concentration; *p*-value for the paired *t*-test (*t*-test: Paired Two Sample for Means).

Storage Conditions	I vs. 1 D	I vs. 3 D	I vs. 5 D	1 D vs. 3 D	1 D vs. 5 D	3 D vs. 5 D
Clearomizer 1 (22 °C)	**0.048**	**0.048**	**0.019**	**0.048**	**0.022**	0.068
Clearomizer 1 (40 °C)	0.099	0.054	**0.023**	**0.0006**	**0.003**	**0.008**
Clearomizer 2 (22 °C)	**0.048**	**0.049**	**0.011**	0.055	**0.004**	**0.0001**
Clearomizer 2 (40 °C)	**0.045**	**0.042**	**0.029**	**0.049**	**0.018**	**0.0043**

I, initial; 1 D, storing for 1 day; 3 D, storing for 3 days; 5 D, storing for 5 days. Bold numbers denote the cases in which differences are statistically significant at 95% confidence level. Clearomizer 1, CE4-type clearomizer; Clearomizer 2, T3S-type clearomizer.

**Table 6:** The influence of the duration of storage on Ni concentration; *p*-value for the paired *t*-test (*t*-test: Paired Two Sample for Means).

Storage Conditions	I vs. 1 D	I vs. 3 D	I vs. 5 D	1 D vs. 3 D	1 D vs. 5 D	3 D vs. 5 D
Clearomizer 1 (22 °C)	**0.013**	**0.018**	**0.018**	0.054	**0.033**	**0.021**
Clearomizer 1 (40 °C)	**0.049**	**0.022**	**0.024**	**0.020**	**0.023**	**0.030**
Clearomizer 2 (22 °C)	**0.045**	**0.023**	**0.041**	**0.017**	**0.047**	0.096
Clearomizer 2 (40 °C)	**0.045**	**0.025**	**0.021**	**0.007**	**0.015**	**0.048**

I, initial; 1 D, storing for 1 day; 3 D, storing for 3 days; 5 D, storing for 5 days. Bold numbers denote the cases in which differences are statistically significant at 95% confidence level. Clearomizer 1, CE4-type clearomizer; Clearomizer 2, T3S-type clearomizer.

**Table 7:** The influence of the duration of storage on Zn concentration; *p*-value for the paired *t*-test (*t*-test: Paired Two Sample for Means).

Storage Conditions	I vs. 1 D	I vs. 3 D	I vs. 5 D	1 D vs. 3 D	1 D vs. 5 D	3 D vs. 5 D
Clearomizer 1 (22 °C)	**0.046**	**0.041**	**0.017**	**0.041**	**0.012**	**0.036**
Clearomizer 1 (40 °C)	**0.027**	**0.009**	**0.008**	**0.015**	**0.013**	**0.049**
Clearomizer 2 (22 °C)	0.064	**0.024**	**0.041**	0.053	**0.041**	0.070
Clearomizer 2 (40 °C)	**0.035**	**0.023**	**0.023**	**0.025**	**0.021**	0.051

I, initial;1 D, storing for 1 day; 3 D, storing for 3 days; 5 D, storing for 5 days. Bold numbers denote the cases in which differences are statistically significant at 95% confidence level. Clearomizer 1, CE4-type clearomizer; Clearomizer 2, T3S-type clearomizer.

## Data Availability

Not applicable.
